# Survey on Continuing Medical Education Needs of Chinese Medical Aesthetic Doctors: A Cross-Sectional Study Based on Questionnaires

**DOI:** 10.1093/asjof/ojaf055

**Published:** 2025-06-06

**Authors:** Zhili Hu, Fanqi Yi, Linggang Kong

## Abstract

Continuing medical education (CME) is essential for aesthetic medicine doctors to remain current and preserve their professional competencies. Nonetheless, the CME needs of Chinese aesthetic medicine practitioners are largely unfulfilled, with minimal investigation into their learning requirements and preferences relative to their professional backgrounds. This gap impedes the efficacy of CME programs. This study explores the landscape of CME among aesthetic medicine doctors in China and assesses their learning requirements, motivations, and preferences across different stages of practice and professional backgrounds. The objective is to provide insights for enhancing the effectiveness of CME programs. A cross-sectional study was conducted among medical aesthetic doctors throughout China. A structured questionnaire, which included sections on demographics, CME status, and learning motivations and preferences, was employed. The questionnaire was distributed electronically to ensure participant anonymity. The data were analyzed using descriptive statistics, multiple-choice analysis, and χ^2^ tests. The authors of this study surveyed 1038 aesthetic medicine doctors from over 1000 cosmetic institutions and revealed that 30.6% had unmet CME needs, particularly in lower tier cities and public hospitals. Key motivations for CME participation included personal interest, opportunities for academic exchange, and maintaining workplace competitiveness, with preferences leaning toward cosmetology associations and online platforms. The highest demand was for training in filler injections, followed by aesthetic laser techniques and surgical procedures. Notably, learning motivations and preferences varied according to years of practice. CME programs should be designed to address the particular needs of aesthetic physicians, thereby enhancing their practice and professional growth. To better achieve CME objectives, training strategies should be tailored according to different practice stages.

**Level of Evidence:** 5 (Therapeutic)

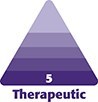

China's medical aesthetics industry has experienced significant growth since the 20th century. By 2024, the market is expected to expand by ∼10%, reaching a value of 345 billion US dollars. Furthermore, it is projected to maintain a compound annual growth rate of 10% to 15% over the subsequent 4 years.^1^ However, this rapid development brings several challenges, including a shortage of professional practitioners, a lack of comprehensive and institutionalized training systems, and the need for enhanced compliance because of concerns over safety, quality, and standardization.^2,3^ The scarcity of well-trained aesthetic medicine doctors is a primary obstacle hindering the industry's healthy development.^2^ Addressing these challenges is crucial for sustaining growth and success.

Young doctors face challenges in their professional development, especially in the constantly evolving field of medical aesthetics.^4^ To meet the industry's development needs, continuous learning and enhancement of professional knowledge and skills are crucial. Continuing medical education (CME) plays a significant role in ensuring that medical professionals stay updated with the latest advancements in their respective fields.^5,6^ A comprehensive CME program is essential for clinicians to maintain high standards of practice.^7,8^ In the field of medical aesthetics, where new techniques and technologies are constantly emerging, ongoing learning is essential for doctors to provide optimal treatment to their patients.^2,3^ Therefore, implementing robust CME activities for aesthetic doctors is a vital strategy to foster talent development in the industry.

Accurately evaluating the learning needs of participants is an essential initial phase in designing educational activities. By implementing well-structured needs assessment, CME have the potential to effectively influence and modify physician behavior.^9^ The learning needs and preferences for CME may vary depending on the doctor's professional background. It is important to identify these variations to design effective CME programs that address their unique needs.^10,11^ Additionally, understanding the motivations of medical aesthetic doctors at different stages of their professional practice is crucial. Doctors with less experience may have different learning interests compared with those with more experience. By identifying these differences, we can develop targeted educational strategies to support medical aesthetic doctors’ growth and development.

Accurately assessing the learning needs of participants is a crucial initial step in designing educational activities. By implementing well-structured needs assessments, CME programs have the potential to effectively influence and modify physician behavior.^9^ The learning needs and preferences for CME can vary based on a doctor's professional background, making it important to identify these variations to design effective programs that address unique needs.^10,11^ Moreover, understanding the motivations of medical aesthetic doctors at different stages of their careers is essential. Less experienced doctors may have different learning interests compared with those with more experience. Identifying these differences allows for the development of targeted educational strategies that support the growth and development of medical aesthetic doctors.

## METHODS

We conducted a cross-sectional survey that included over 1038 medical aesthetic practitioners from >1000 institutions across various cities. First-tier cities, such as Beijing, Shanghai, Guangzhou, and Shenzhen, were distinguished by their advanced economic development and international prominence. In contrast, second- and third-tier cities, including Chengdu, Hangzhou, and Wuhan, although less developed, demonstrated substantial economic growth. Other cities were categorized as fourth and fifth tiers, characterized by their small size, weak economic foundations, and inadequate transportation facilities. The questionnaire encompassed doctors with a range of professional titles categorized according to their titles and levels of experience. Junior physicians were recent graduates or those undergoing residency training. Intermediate physicians possessed several years of clinical experience and had attained the “Attending Physician” title following the successful completion of relevant examinations. Senior physicians were distinguished by extensive experience, holding titles such as “Associate Chief Physician” or “Chief Physician,” which required rigorous evaluations and significant contributions to research or teaching. We utilized a self-assessment questionnaire to collect data for the CME survey (Supplemental Figure 1). The data collection process spanned from March 2023 to September 2023, during which the questionnaire was distributed online. Practitioners were identified and selected through a combination of professional networks, industry databases, and relevant medical associations to ensure a representative sample of the medical aesthetics field. Initial contact was established through email, where participants were informed about the survey's purpose and significance. To encourage doctors to complete the questionnaire, an incentive was offered that would award respondents with points that could be redeemed for educational material rewards (eg, books and stationary). The collected data were analyzed using descriptive statistics, multiple-choice analysis, and χ^2^ tests. All data analyses were conducted using SPSS 20.0 software (IBM SPSS Statistics, IBM Company, Armonk, NY).

The questionnaire was divided into 3 sections (Supplemental Figure 1): demographics (6 questions), status survey (6 questions), and needs survey (5 questions). Questions on CME needs were multiple choice and not ranked choice. The percentages represent the number of times an option was selected divided by the total number of valid responses.

## RESULTS

### Demographic Information of Chinese Aesthetic Doctors

The study involved 1038 responses out of 1221 distributed questionnaires, achieving an 85% response rate among ∼1000 medical institutions. Of the respondents, 43.4% were male and 56.6% female. Participants predominantly practiced as plastic surgeons (58.4%) and dermatologists (37.9%), with other specialties such as traditional Chinese medicine and stomatology comprising 3.6%. Physicians’ titles were distributed as intermediate (58.2%), junior (23.5%), and senior (18.3%). Regarding experience, 30.7% had practiced for 5 to 10 years, 28.5% for 3 to 5 years, 25.2% for <3 years, and 15.6% for >10 years. A significant proportion, 63.3%, were in first-tier cities, whereas 33.8% were from second- and third-tier cities, and 2.9% were from fourth- and fifth-tier cities. Private institutions employed 75.5% of doctors, whereas 24.5% worked in public institutions (Table 1).

**Table 1.** The Background of the Survey Respondents and the Factors Influencing the Unmet Learning Needs of Chinese Aesthetic Doctors

	The unmet learning needs				
Variables		Total	No. of people with unmet needs	%	*P*-value
Gender	Male	450	136	30.2	
	Female	588	182	69.8	.800
Majors	Plastic surgery	606	182	30.0	.883
	Dermatology	394	124	31.4	
	Others	38	12	31.6	
Doctor titles	Junior physicians	244	72	29.5	.795
	Intermediate Physician	604	190	31.5	
	Senior physician	190	56	29.5	
Years of practice	<3	262	83	31.8	.834
	3-5	296	90	31.2	
	5-10	318	100	31.5	
	>10	162	45	29.7	
City of work	First-tier cities	657	173	26.3	.000***
	Second- and third-tier cities	351	133	37.9	
	Fourth- and fifth-tier cities	30	12	40.0	
Hospital attributes	Public hospital	254	91	35.8	.039*
	Private hospital	784	227	29.0	

***P < .001, *P < .05.

### Current Situation of Continuing Medical Education for Aesthetic Physicians in China

**Figure 1.**
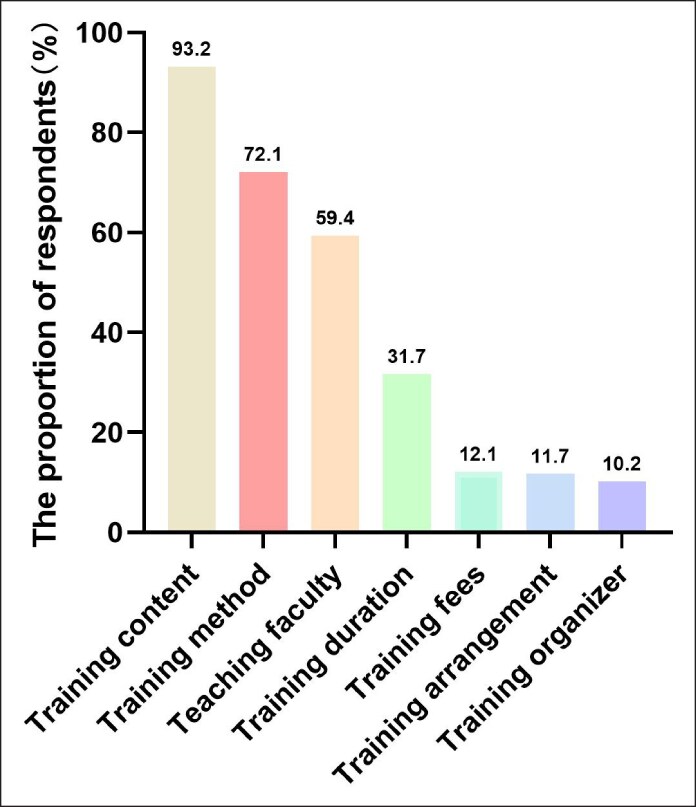
Factors affecting satisfaction with continuing medical education for Chinese aesthetic doctors.

The findings indicated that 30.6% (318 out of 1038) of doctors experienced unmet CME needs. Rates of unmet need were notably higher in second- and third-tier cities (37.9%) and fourth- and fifth-tier cities (40.0%) compared with their first-tier counterparts (26.3%, *P* < .001). Employment in public hospitals was associated with higher unmet needs (35.8%) relative to private hospitals (29.0%; *P* = .039). Concerning the effectiveness of CME, the most frequently identified factors were training content, training methods, and teaching faculty, accounting for 93.2%, 72.1%, and 59.4%, respectively (Figure 1).

### Learning Requirements for Continuing Medical Education in Chinese Aesthetic Doctors

**Figure 2.**
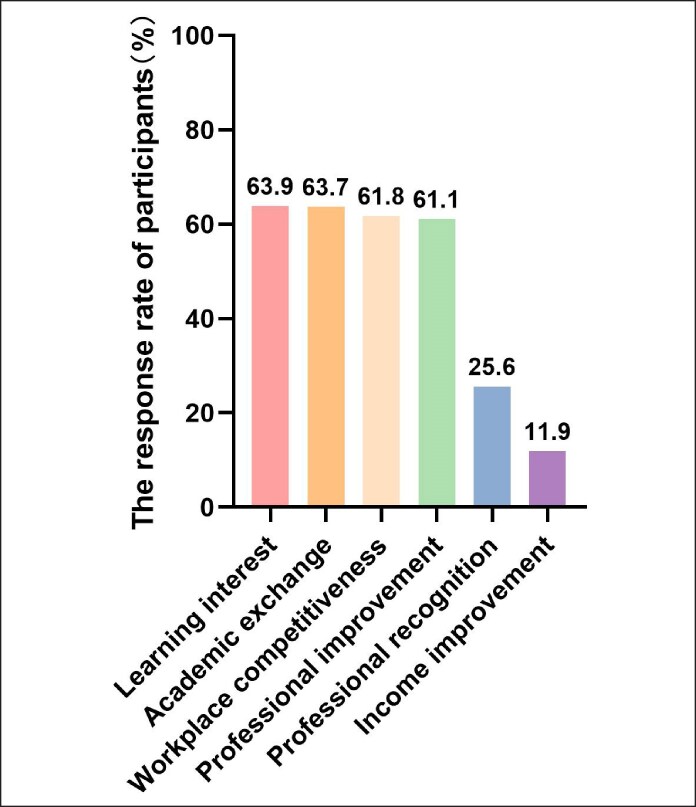
Factors motivating engagement in continuing medical education for Chinese medical aesthetic doctors.

Motivations for engaging in CME varied among aesthetic physicians. The primary drivers included personal interest in learning (63.9%), opportunities for academic exchange (63.7%), and maintaining workplace competitiveness (61.8%). Approximately one-fourth (25.6%) of doctors engaged in such training for professional recognition, and 11.9% of doctors aimed to increase their personal income (Figure 2).

Motivation also differed based on years of practice (*P* = .003). Doctors with <3 years of experience primarily participated in CME because of personal interest, accounting for 24.6%. Those with 3 to 5 years of experience aimed to enhance professional competitiveness, representing 23.3%. Physicians with 5 to 10 years and those with over 10 years of experience engaged in CME for opportunities for academic exchange, with proportions of 22.3% and 22.4%, respectively (Table 2).

**Table 2.** Factors Motivating Engagement in Continuing Medical Education for Chinese Medical Aesthetic Doctors, Based on Years in Practice

Variables	Individual learning interest	Workplace competitiveness	Academic exchange	Income improvement	Professional improvement	Professional recognition	*P*-value
	*n* (%)	*n* (%)	*n* (%)	*n* (%)	*n* (%)	*n* (%)	
Years of practice							
<3	176 (24.6%)	165 (23.1%)	143 (20.0%)	9 (1.3%)	167 (23.4%)	54 (7.6%)	
3-5	186 (21.7%)	200 (23.3%)	191 (22.3%)	38 (4.4%)	174 (20.3%)	69 (8.0%)	.003**
5-10	201 (21.8%)	189 (20.5%)	215 (23.3%)	35 (3.9%)	192 (20.8%)	89 (9.7%)	
>10	101 (20.3%)	88 (17.7%)	112 (22.5%)	42 (8.4%)	101 (20.3%)	54 (10.8%)	

**P < .01.

**Figure 3.**
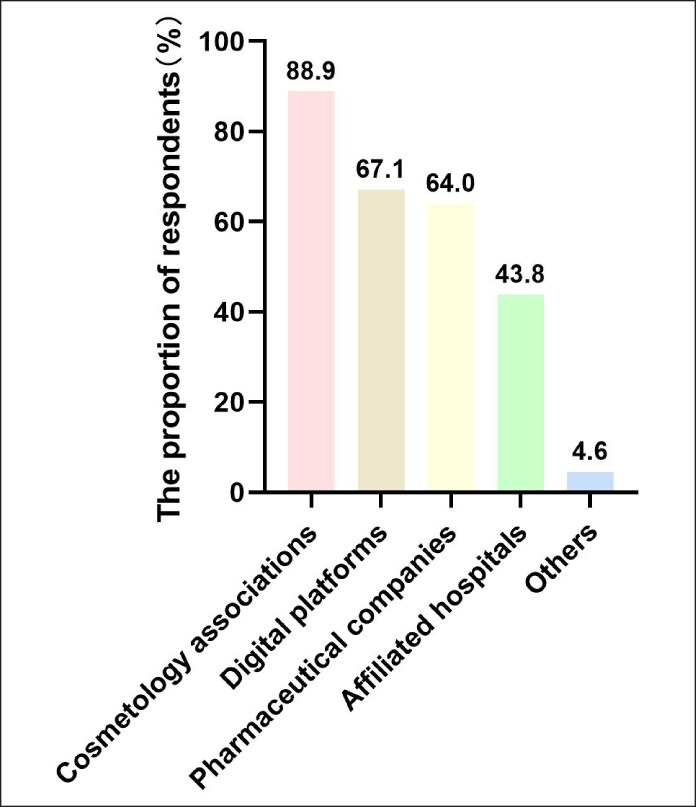
Preferred learning avenues in continuing medical education for Chinese medical aesthetic doctors.

Chinese aesthetic doctors showed a preference for CME channels dominated by cosmetology associations (88.9%) and online learning platforms (67.1%). Pharmaceutical company-led activities accounted for 64.0%, followed by CME initiatives from university-affiliated hospitals, which comprised ∼43.8% (Figure 3).

**Figure 4.**
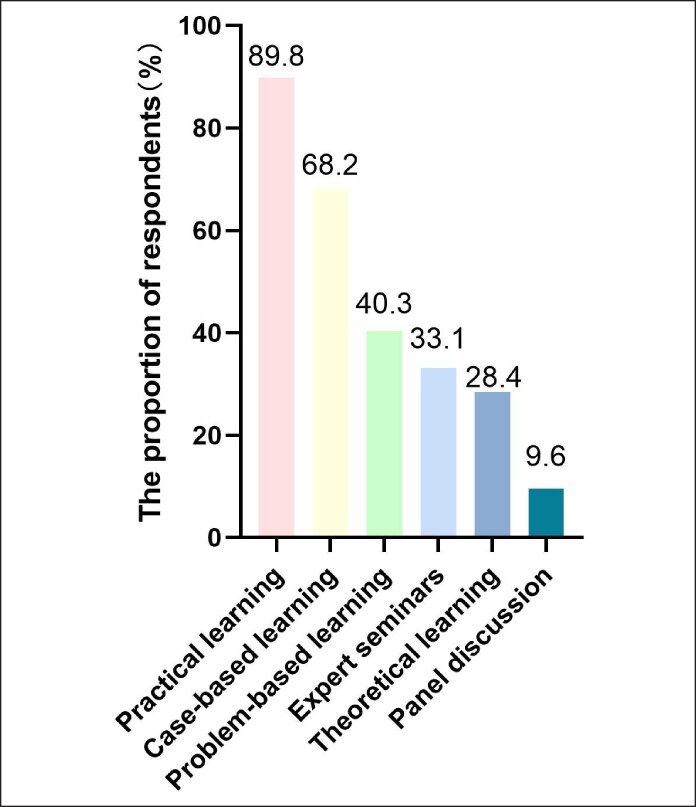
Preferred learning methods in continuing medical education for Chinese medical aesthetic doctors.

The combination of theoretical and practical learning was highly favored by 89.8% of doctors, alongside case-based learning approaches (68.2%). Expert seminars were also favored by 40.3% of the participants. Additionally, preferences for theoretical learning, group discussions, and online learning stood at 33.1%, 28.4%, and 9.6%, respectively (Figure 4).

### Training Content for Continuing Medical Education

**Figure 5.**
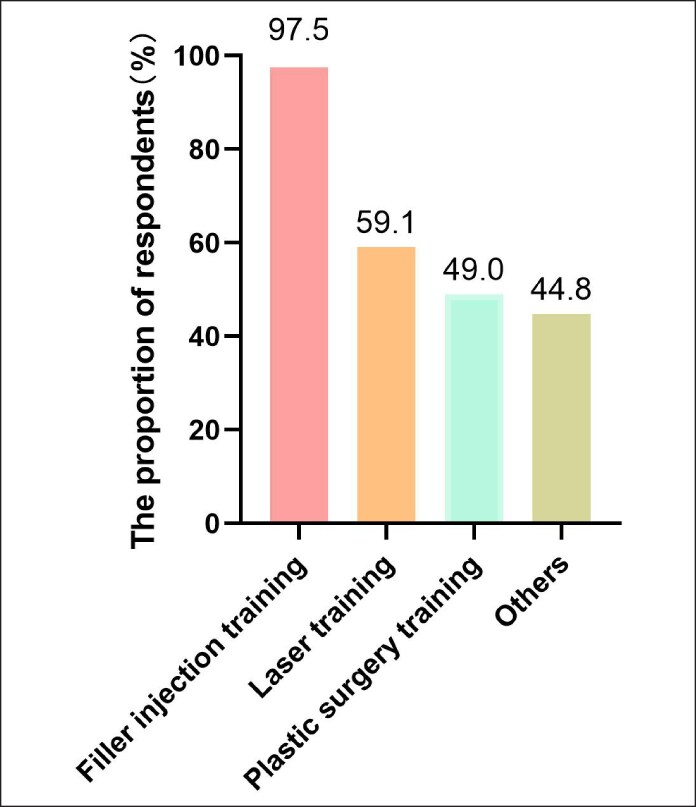
Preferred educational topic in continuing medical education for Chinese medical aesthetic doctors.

The demand for CME content was heavily skewed toward filler injections, with 97.5% of respondents expressing interest, followed by aesthetic laser techniques (59.1%) and surgical procedures (49.0%). Additionally, other antiaging treatments, such as chemical peels, garnered interest from ∼44.8% of participants (Figure 5).

Learning modules were categorized to address varying needs, with an emphasis on injection techniques (73.6%), facial aesthetic design (64.6%), and facial anatomy (57.1%). Management of complications, facial assessment, communication skills, and knowledge of filling products accounted for 33.7%, 31.3%, 27.3%, and 8.9%, respectively. Variations in learning needs were prominently driven by practice duration (*P* < .001). Doctors with <3 years of practice focused primarily on learning injection techniques, accounting for 71.2%. Those with 3 to 5 years of practice preferred studying anatomy courses, accounting for 37.7%. Doctors with 5 to 10 years and >10 years of practice tended to focus on aesthetics, accounting for 23.1% and 30.8%, respectively (Table 3).

**Table 3.** Preferred Continuing Medical Education Educational Topics for Chinese Medical Aesthetic Doctors, Based on Years in Practice

Variables	Learning content							
	Anatomy courses	Injection Techniques	Aesthetics	Facial assessment	Communication skills	Complication management	Injectable product characteristics	*P*-value
Years of practice								
<3	32 (8.7%)	262 (71.2%)	25 (6.8%)	9 (2.4%)	10 (2.7%)	12 (3.3%)	18 (4.9%)	
3-5	260 (37.7%)	192 (27.8%)	102 (14.8%)	40 (5.8%)	40 (5.8%)	40 (5.8%)	16 (2.3%)	
5-10	149 (14.7%)	177 (17.4%)	234 (23.1%)	160 (15.8%)	86 (8.5%)	180 (17.8%)	28 (2.7%)	<.001***
>10	152 (15.1%)	133 (13.2%)	310 (30.8%)	116 (11.5%)	147 (14.6%)	118 (11.7%)	31 (3.1%)	

***P < .001, *P < .05.

## DISCUSSION

The shortage of professional doctors in China's medical aesthetics industry has become a significant obstacle to its progress. The medical education system in China comprises medical colleges, graduate medical education, and CME.^12^ Medical aesthetic doctors are required to accumulate CME credits through conferences, training courses, and online education, ensuring they remain current with medical advancements.^13^ However, CME in medical aesthetics faces challenges, such as the lack of standardized guidelines, faculty shortages, and financial constraints.^14^ Furthermore, individualized CME activities are necessary to meet diverse demands that vary by professional fields and stages of development.

Our research indicated that the demand for CME among medical aesthetic doctors remains high, with unmet educational needs particularly prevalent in fourth- and fifth-tier cities and public hospitals. Understanding regional and institutional disparities is crucial for tailoring ongoing education programs to efficiently address specific needs. Additionally, varied motivations drive doctors to pursue CME, including personal interest, workplace competitiveness, and academic exchange, depending on their career stage.

Medical aesthetic doctors require additional or specialized motivation to pursue CME. Understanding their learning motivation can aid in developing targeted programs to meet their diverse needs. Our research demonstrated that motivation varied among doctors. Junior doctors were driven by personal interest in learning, whereas those with 3 to 5 years of experience sought to enhance their competitiveness. Doctors with longer years of practice (>5 years) were more concerned with opportunities for academic exchange.

Previous studies have demonstrated that professional doctors often employ nonsystematic learning strategies because of the demands of clinical practice.^15^ Industry associations and online platforms are popular CME channels.^16^ However, company-led activities raise concerns about dependency and resource allocation.^17^ Therefore, collaborations with reasonable regulations are necessary to ensure content quality and objectivity.

To maximize the effectiveness of medical education, diverse methods, such as combining theory with practice and implementing case-based learning, are preferred.^18^ CME program content should align with clinical needs and rapidly evolving industry developments, with an emphasis on non-invasive procedures like injections and phototherapy.^19,20^ Our findings underscore a pressing need for training in injection techniques, with interest varying by experience level: doctors with <3 years of practice focusing on injection techniques, those with 3 to 5 years concentrating on anatomy, and doctors with more extensive experience prioritizing complication management and communication skills.

This study employed a questionnaire survey, with limitations including selection bias and sample size constraints. Predefined answer options might have limited respondents’ ability to fully express their needs, and self-reporting biases may have affected accuracy. Additionally, offering incentives to participants could have contributed to selection bias. Future efforts should focus on improving survey design to capture more comprehensive feedback.

## CONCLUSIONS

CME plays a crucial role in the professional development of medical aesthetic doctors in China. Our study identified key challenges, such as regional disparities and diverse training needs, which impact the effectiveness of CME. The research highlights that doctors prioritize learning injection techniques, facial aesthetic assessment, and facial anatomy, with motivations and preferences varying across different career stages. Tailored and well-regulated CME programs are essential for addressing unmet educational needs and enhancing training effectiveness.

## Supplementary Material

ojaf055_Supplementary_Data
