# Supplementary figures and images for: Survey on Continuing Medical Education Needs of Chinese Medical Aesthetic Doctors: A Cross-Sectional Study Based on Questionnaires
Source: Aesthet Surg J Open Forum. 2025 Jun 6;7:ojaf055. doi: 10.1093/asjof/ojaf055 (PMC12290396; doi:10.1093/asjof/ojaf055)

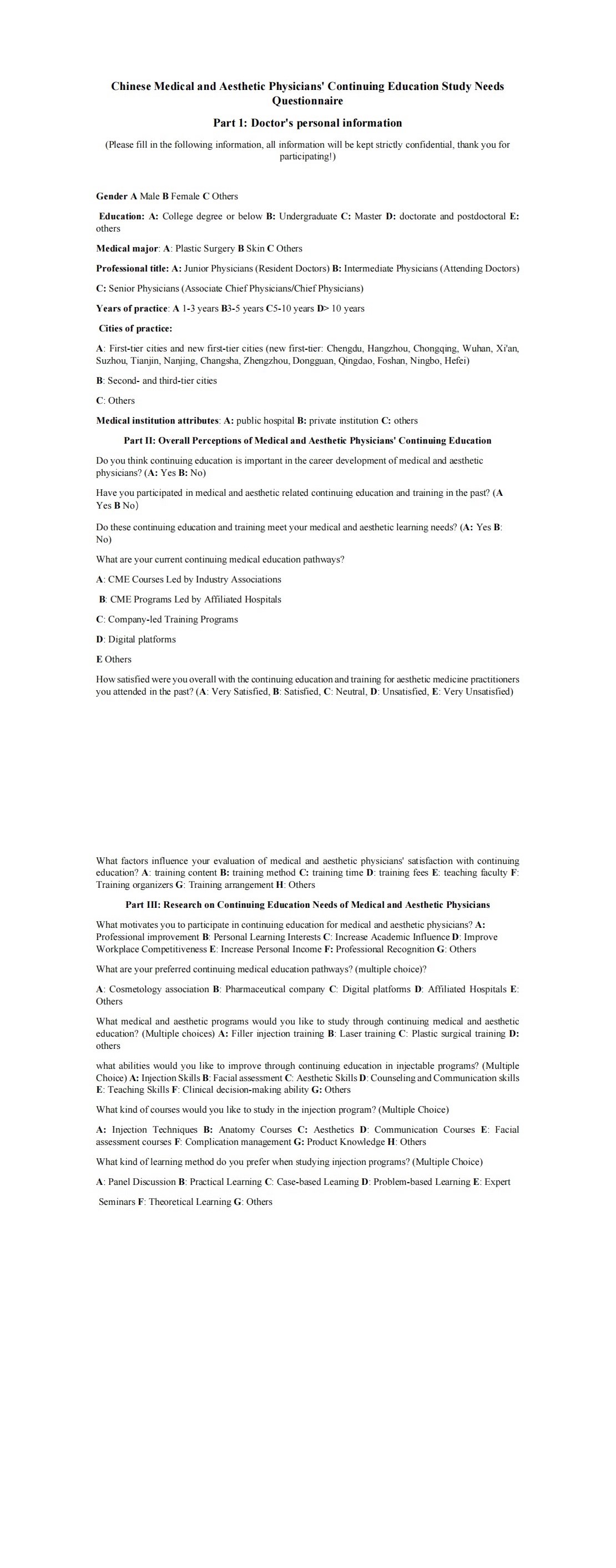

Supplement: ojaf055_Supplementary_Data [file ojaf055_supplementary_data.jpeg]
